# Cutaneous and Respiratory Lesions in Bushfire-Affected Koalas

**DOI:** 10.3390/vetsci10110658

**Published:** 2023-11-16

**Authors:** Chloe Baek, Lucy Woolford, Oliver Funnell, Jennifer McLelland, Stuart Eddy, Tamsyn Stephenson, Natasha Speight

**Affiliations:** 1 School of Animal and Veterinary Sciences, Faculty of Sciences, Engineering and Technology, University of Adelaide, Roseworthy, SA 5371, Australialucy.woolford@adelaide.edu.au (L.W.); tamsyn.stephenson@adelaide.edu.au (T.S.); 2 Zoos South Australia, Frome Rd., Adelaide, SA 5000, Australia; ofunnell@zoossa.com.au (O.F.); jmclelland@zoossa.com.au (J.M.); 3 The Austin Vet Specialists, Adelaide, SA 5031, Australia; stuarteddy@theaustin.vet

**Keywords:** burn, bushfire, histopathology, koala, Phascolarctidae, smoke, wildfire

## Abstract

**Simple Summary:**

In the summer of 2019–2020, multiple states of Australia experienced catastrophic bushfires, with koalas being the main wildlife species rescued. Many suffered burns which affected all parts of their body, but particularly their footpads. It can be more difficult to assess the severity of burns in animals than in humans due to the variation in their foot morphology (footpads, hooves, paws), and because fur covers the majority of their body. Here, we describe the clinical and microscopic findings of burns in koalas, following biopsies obtained from animals that were euthanised on welfare grounds after rescue from bushfire. We also describe microscopic findings in the lungs of koalas due to smoke inhalation. These findings will assist veterinarians involved with triaging koalas affected by burns and help predict if they are candidates for rehabilitation or if their prognosis is too poor.

**Abstract:**

In the wake of increasingly frequent bushfires emerging as a threat to wildlife worldwide, koalas have notably been the most rescued species in Australia. However, our understanding of burns and their severity in koalas is limited; hence, this study investigated the histopathological features and depth of burns in koala skin, as well as the presence of smoke-induced respiratory tract damage. In four bushfire-affected koalas that had been euthanised on welfare grounds, skin burns in various body regions were scored based on clinical appearance as superficial, partial thickness, or full thickness. Histological sections of affected regions of skin were assessed as Grades I–IV and showed that furred regions on the ear margins and dorsum were histologically more severe, at Grade III, compared with the clinical score. There was a similar finding for footpad burns, which were the most common body region affected. In the respiratory tract, pulmonary oedema and congestion were evident in all koalas. Overall, the results highlight that cutaneous burn lesions on furred and palmar/plantar surfaces can have higher severity based on the burn depth than is clinically apparent. Therefore, there is a need to consider this when developing treatment plans and establishing prognosis for burnt koalas at triage, as well as that a high likelihood of pulmonary oedema exists.

## 1. Introduction

In the summer of 2019–2020, multiple states of Australia experienced catastrophic bushfires affecting over 3 billion native vertebrate animals [1]. Large-scale wildlife rescue efforts were undertaken, and, in Victoria, koalas (*Phascolarctos cinereus*) accounted for 75% of rescued animals [2]. On Kangaroo Island, South Australia, koalas were triaged and their burn severity was recorded, along with other clinical and demographic data [3]. In animals, there can be diagnostic challenges with scoring thermal wounds [4,5], as human-based scales have to be adapted [6] and may not accurately score the burns on either furred skin or the variable palmar and plantar regions of different species, e.g., hooves or paws.

In dogs and cats, a clinical scoring system of 1–3 (superficial, partial thickness, full thickness) is used [6], based on the scale used in humans [7]. The severity of the burn may be determined by considering the clinical appearance of the wound, where partial-thickness and full-thickness injuries will be readily apparent after the burn develops a thick leathery surface of necrotic tissue [4]. Superficial burns are restricted to the epidermis and can heal without leaving a scar, and partial thickness may rapidly heal via re-epithelialisation. Full-thickness burns destroy all cutaneous structures, resulting in hypertrophic scarring and potential deformation [5]. However, due to the intricacy of the dynamic changes that occur in the cellular and parenchymal parts of skin following a burn, clinically determining its depth with accuracy is difficult, particularly in furred animals.

Fire victims also often have smoke inhalation damage in both the upper and lower airways, primarily causing pulmonary oedema. This may result in signs of respiratory distress, such as tachypnoea, dyspnoea, open-mouth breathing, or frothy nasal discharge [8]. Protein loss into the oedema fluid can potentially lead to hypoproteinemia and when combined with hypovolemia could result in cellular hypoxia, simultaneously affecting many organs, such as lungs, liver and kidney [5]. In addition, soot is commonly found in oral and nasal cavities and also lower down the respiratory tract [4].

In koalas, previous reports of burns in bushfire-rescued animals highlight smoke inhalation and burns to the footpads [3,9,10], which are highly keratinised on both the fore and hind limbs due to their quadrupedal gait when walking. There have also been reports of damaged or lost claws in koalas with footpad burns [3]. Burns to the face are also common, and may affect the eyes, nose, lips and ears [3]. Burns to these body regions have importance in feeding and climbing behaviours, which are essential for koala survival. Accurate assessment of burn severity in koalas directs decision making, including euthanasia on animal welfare grounds, and for those with good prognosis, choice of treatments in the rehabilitation process [2,3].

Ultimately, only skin histology can accurately assess the degree of the burn by characterising the pathological changes in the epidermis, dermis and hypodermis as reported in experimental animal models [11], but this has not previously been reported for koalas euthanised after bushfire. Hence, this study aimed to describe histopathologic changes caused by bushfire burns to the skin of the footpads, furred body regions and claws of koalas to improve our understanding of burn severity in this iconic wildlife species. Furthermore, we describe the effect of smoke inhalation on the tissues of the respiratory tract.

## 2. Materials and Methods

On 24 January 2021, a bushfire in Cherry Gardens in the Mount Lofty Ranges, South Australia, burnt through 2700 ha of scrub and grassland. Four wild koalas that were euthanised on welfare grounds were received the following week and stored frozen for necropsy examination. Due to claw abnormalities being noted, prior to necropsy, radiographs were taken of the fore and hind digits of three of the koalas. Age was estimated according to the tooth wear class (TWC) method on the basis of dental wear of the fourth upper premolar [12]. Body condition score (scale of 1 to 5, poor to excellent) was based on palpation of scapular muscle mass [13], and the body weight of each koala was recorded.

At necropsy, burn depth was scored as superficial, partial thickness or full thickness based on clinical appearance. This scoring system aligned with that used in burn severity assessment in Kangaroo Island koalas, as reported in Dunstan et al. (2021), based on the system used in dogs and cats [6]. Samples of affected skin and respiratory tract tissues were collected into 10% neutral buffered formalin, processed routinely and stained with haematoxylin and eosin for microscopic examination. Several digits with claw abnormalities were also collected and decalcified prior to histological processing, with sections focussed on the distal phalanx.

Histologically, cutaneous burn lesions were scored as Grade I (first degree—superficial epidermis affected only), Grade IIa (second degree—superficial; total epidermis excluding germinative/basal keratinocyte layer, and partial dermis), Grade IIb (second degree—deep; total epidermis and partial dermis, excluding adnexal structures), Grade III (third degree—epidermis and dermis, including adnexal structures) and Grade IV (fourth degree—epidermis, dermis, subcutis and underlying tissues) [4].

## 3. Results

Of the four bushfire-affected koalas examined, three were female and one was male. A summary of the tooth wear class, estimated age, body condition score and weight of koalas is shown in Table 1.

### 3.1. Necropsy Findings

The footpads were the most consistently burnt part of the body of the examined koalas.

**Figure 1:**
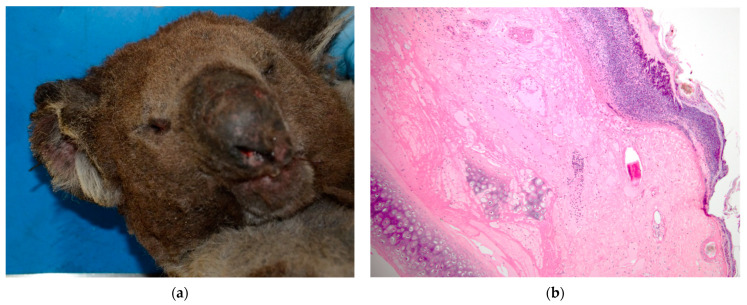
Koala K21-047 ear margins: (**a**) photograph of entirely singed ear margins scored as superficial thickness at necropsy, with facial fur blackened with soot and nostrils superficially burnt; (**b**) histological lesions of ear margin scored as Grade III. The epidermis is lost and replaced with degenerate neutrophils embedded within a proteinaceous coagulum, with coagulative necrosis extending to the deep adnexal structures.

For koala K21-047, the fur was singed all over the body, and the nostrils and pinna margins recorded as superficial-thickness burns (Figure 1a). Bilateral burn lesions of superficial thickness were recorded over the dorsal pelvic regions with smaller areas (approx. 3 × 3 cm) of partial thickness over the iliac crests. The entire dorsum was singed and covered in soot particles, and the tip of the penis was erythematous.

The palmar surfaces were intact, with the claw of digit IV on the left having a blunted tip, and the claws of digits III and IV on the right having increased erythema on the concave surface. The left distal plantar surface of the footpad and digits I, IV and V were scored as having superficial-thickness burns. The entire right plantar surface, including all digits, was affected by superficial burns with partial-thickness burns on the lateral aspect. In the respiratory tract, serosanguinous froth and soot particles were present in the trachea, and congestion affected all lung lobes, particularly the right middle and caudal lobe margins.

**Figure 2:**
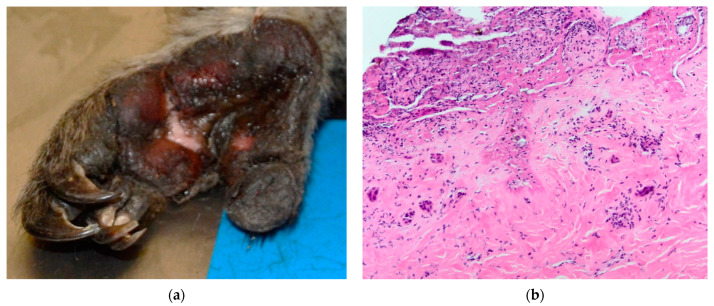
Koala K21-048 left plantar surface: (**a**) scored as partial thickness at necropsy; (**b**) histological examination showed Grade III burn with full-thickness epidermal necrosis, infiltration of leukocytes, and exudation of fibrin. The superficial to mid-dermis was diffusely pale and blood vessel plexi and adnexa showed loss of cellular detail, condensation of nuclear chromatin and pyknosis.

Koala K21-048 presented with soot particles around the nostrils and mouth and an erythematous nasal planum. Soot particles were also noted on the chest and rump. The claw of digit III on the right fore limb had a blunted tip. The distal region of digit I on the right hind showed sloughing of the superficial skin, whilst superficial burns covered the entire left plantar surface, including the digits, with regions of partial-thickness burns on the distal, proximal and lateral plantar surface (Figure 2a). The lung lobes were congested in the dorsal aspects.

Koala K21-050 was recorded with soot around the nostrils and mouth, and on the chest, upper fore limbs, dorsum of the trunk and rump. The central region of the right palmar surface, including that of digits III–V, was noted to have superficial-thickness burns. Similarly, the left palmar surface of digits III–V showed superficial burns, with proximal involvement of the palm. The claw of digit III showed a blunted tip, and the claw of digit IV was missing. The left plantar surface showed superficial burns on the lateral half with a small localised partial-thickness lesion centrally. The distal phalanx of digit V was fractured and displaced at the base of the claw. The entire right plantar surface showed superficial burns which extended to the plantar surface of digits II–V. The lung lobes were congested, particularly on the margins.

Koala K21-057 presented with superficial burns to all four footpads. There were small regions of superficial burns on the palmar surface of left digits I and III–V, whilst the entire right palmar surface of digit IV was superficially burnt with small regions on digits III and V. The tip of the claw of digit V was blunted. The left plantar surface of digit V showed superficial-thickness burns, with a small region on digit IV. The right lateral plantar surface skin was sloughed and there were small regions of superficial-thickness plantar surface burns on digits II–V and the base of digit I. The lung lobes showed areas of congestion.

### 3.2. Histopathology of Cutaneous Lesions

**Figure 3:**
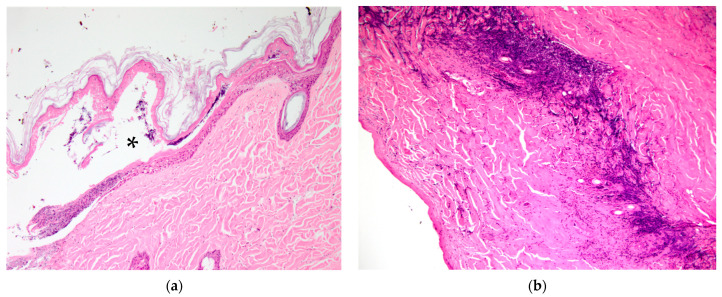
Koala K21-047 dorsal skin over the iliac crest region: (**a**) Grade IIa lesion, with intra- or sub-epidermal vesicle formation shown by asterisk; (**b**) Grade III lesion with a band of neutrophils present between devitalised and vitalised tissue.

Burn Grades I–III were observed in skin sampled from affected koalas in the selected sites (Table 2), with transition across sequential grades observed at most sites. Grade I and IIa lesions were most commonly observed adjacent to lesions of higher grades. Grade I lesions were characterised by cytoplasmic vesiculation of superficial keratinocytes and mild hyperkeratosis and were seen in one koala only (right fore footpad, K21-057). Grade IIa lesions were characterised by occasional intra- or subepidermal vesicle formation with accumulation of proteinaceous material and basophilic granular debris (Figure 3a, K21-047 furred dorsal skin over iliac crests), and epithelial and follicular coagulative necrosis, with variable fibrinosuppurative inflammation.

In Grade IIb lesions, there was diffuse coagulative necrosis of all layers of the epidermis characterised by loss of cellular and nuclear detail and superficial dermal pallor and variable basophilia, with only deep adnexal structures remaining viable. Fibrin exudation was variable. Grade IIb lesions were the least commonly observed, being found on one footpad only.

**Figure 4:**
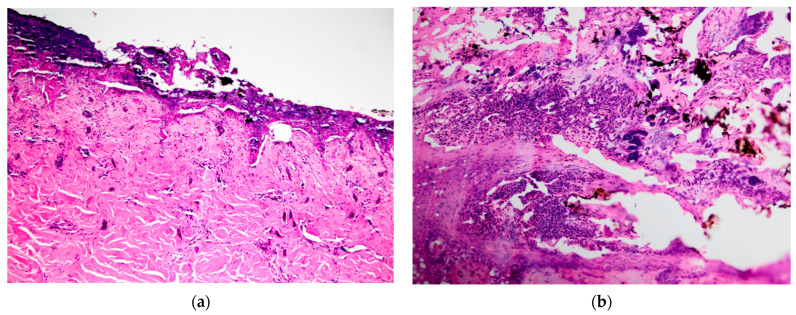
Koala plantar surfaces: (**a**) histological lesions of K21-048 left hind lateral footpad showing coagulative necrosis in the dermis and soot particles; (**b**) histological lesions of K21-047 right hind lateral footpad showing bacterial colonies in the epidermis, indicative of secondary infection. Soot particles were also present.

Grade III lesions were the most common, being seen in the footpads of three koalas and the pinna and dorsal skin over the left and right iliac crest regions in one koala (K21-047) (Figure 3b). Grade III lesions were typified by coagulative necrosis of the epidermis, dermis and adnexal structures, extending to the subcutis. Variable fibrin exudation (Figure 2b, K21-048 left plantar surface), vascular thrombosis and subcutaneous vasculitis were often seen. In some regions, a band of neutrophilic inflammation could be seen between devitalised and non-devitalised tissue (Figure 3b, K21-047 skin over iliac crests). The epidermis was frequently lost, with variable surface exudation of neutrophils within a proteinaceous coagulum, being most notable in the pinna lesion. In addition to these changes, the pinna lesion showed striking extension of subcutaneous fibrin exudation and haemorrhage down the inner and outer surface of pinna from the tip (Figure 1b, K21-047 pinna margin), a change not observed at other sites. Footpads frequently showed surface epidermal brown-black variable refractile pigment material, interpreted as soot/carbon particles and necrotic debris (Figure 4a, K21-048). Bacterial cocci were often seen in regions of suppurative inflammation, indicating secondary bacterial infection of burns (Figure 4b, K21-047). Grade IV lesions were not observed.

### 3.3. Assessment of Claws

**Figure 5:**
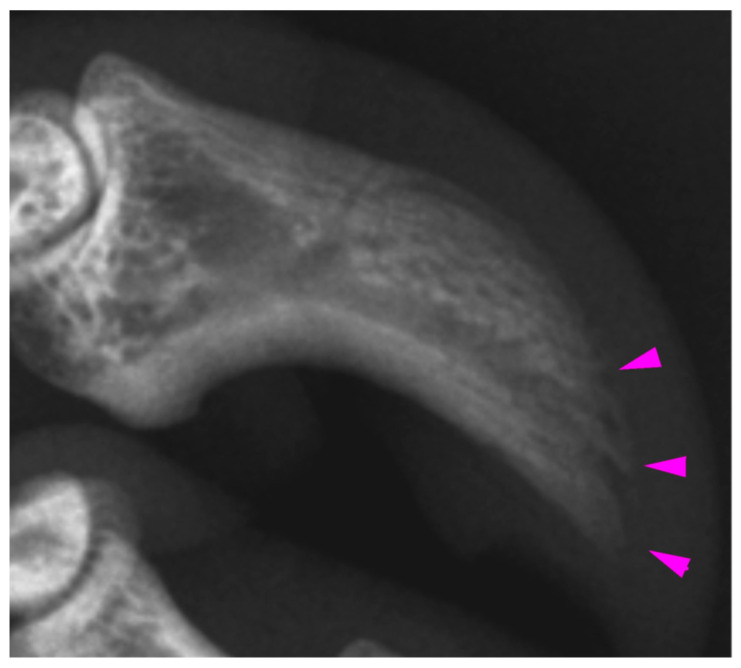
Mild acro-osteolysis (arrows) of the distal phalanx of right fore digit IV in K21-047. This claw showed erythema at necropsy but was not examined histologically.

Radiologic examination of the digits of K21-047 showed mild acro-osteolysis of the distal phalanx of digits III and IV (Figure 5) of the right fore limb, with the claw of digit IV having previously been noted at necropsy to have mild erythema on the concave surface. In both hind limbs, digits II and III showed irregular lytic cortices, with more marked changes distally. On the right hind, digit III and to a lesser extent, digit II, also showed sclerosis of the medullary cavity. Digit V did not show any radiographic changes despite having superficial plantar burns present at necropsy. This digit was selected for histological examination but was unremarkable. For K21-048, the distal phalanges of the hind feet showed mild lytic changes to the distal phalanges, most notable in left digits II and IV. The digits of the fore limbs were unremarkable, despite a blunted claw tip being noted at necropsy in right fore digit III. This digit was selected for histological examination but was unremarkable. For K21-050, the claw loss of left fore digit IV and the fracture of distal phalanx V on the left hind were the only radiologic findings. Histologically, associated with the claw loss from left fore digit IV, there was necrosis of the nailbed epithelium and underlying dermis and exudation of proteinaceous material in the dermis overlying the distal phalanx, indicating necrosis. Soot was present over the exposed surface of the ungual process. There were no detectable histological abnormalities found in left fore digit III despite a blunted claw tip at necropsy.

### 3.4. Histopathology of the Respiratory Tract

**Figure 6:**
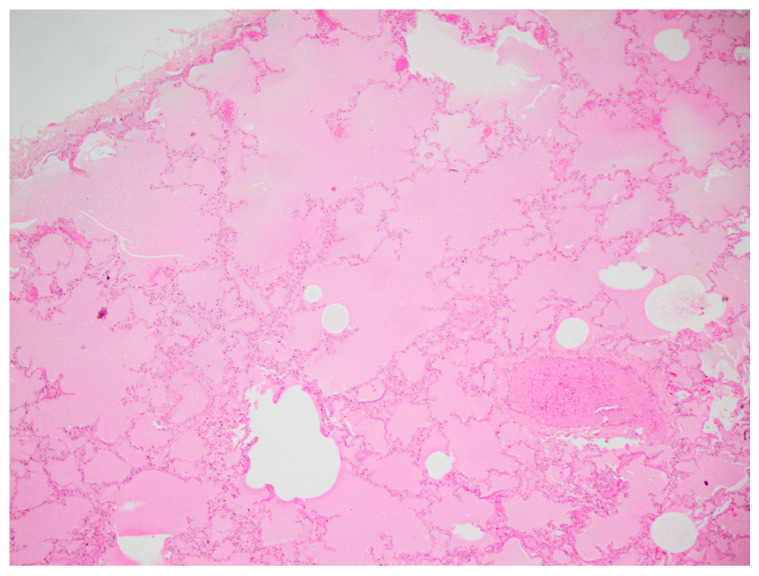
Histological images of lung tissue of koala K21-048. Congestion of lung from protein-rich fluid present in the alveolar lumina, indicating pulmonary oedema.

In the lungs of all four koalas, diffuse alveolar congestion was observed with variable flooding of airways by translucent eosinophilic protein-rich oedema fluid, which was interpreted as alveolar injury and increased vascular permeability (Figure 6). Lymphatic dilation was present in the pleurae, and alveolar macrophages showed foamy cytoplasm as well as refractile brown granular particles consistent with soot. Tracheal mucosa was unremarkable, but intraluminal soot particles were present.

## 4. Discussion

Our study describes the characteristics of burn injuries to the skin of both the furred and unfurred body regions, and the respiratory tract, in euthanised koalas rescued from bushfire. The most common and severe areas of burn injury in koalas occurred in the plantar footpad skin of the hind limbs, likely due to their descent from trees to the scorched ground. Clinical assessment of burn depth often underestimated burn severity, particularly in the furred skin regions, according to the histopathologic assessment, which found that more severe damage of deeper cutaneous structures had occurred. This demonstrates the difficulty in assessing burns in animals, as reported by previous studies [4,5]. Damage to the nailbed of claws was difficult to determine, despite previous reports of claw loss in koalas rescued from bushfires [3,10]. In addition, the finding of pulmonary oedema in all four koalas shows the importance of considering smoke inhalation in treating and rehabilitating wildlife.

The clinical appearance of the burns of the four koalas varied from superficial to partial thickness, and microscopically, between Grade I and III. The most notable differences between clinical and histopathological scores occurred in the furred regions of koala K21-047. At necropsy, superficial burns on the ear margins were observed; however, the histopathological evaluation was Grade III. Similarly, for the dorsal skin over the left and right iliac crest regions of the same animal, the histopathological grading was Grade III, while the clinical assessment determined superficial- to partial-thickness burns. It is possible that the burn severity in these furred regions is difficult to visualise at clinical examination, and that the dense fur increases the heat convection, worsening the burn damage to deeper layers of the skin. Also, partial-thickness burns in dogs and cats can often create an exterior leathery eschar over several days. This makes it difficult to distinguish partial-thickness from full-thickness burns until this outer partial-thickness eschar separates [6], which could have happened in the case of K21-047’s dorsal skin. The koalas were received within a week of the beginning of the bushfire, suggesting that the full extent of their burns may not have yet been visible. In future, fur around burns could be carefully clipped to visualise the area better [4] and the animals receiving treatment monitored over time for worsening of lesions. However, in humans, it has previously been found that even when conducted by experienced burn surgeons, clinical assessment of burn depth has only 50–80% accuracy [11].

For the plantar surfaces, the clinical assessment score of burn severity was lower than that established with histopathologic grading, which was Grade III or transitioning from Grade II in most cases. The lateral plantar surface was particularly prone to deep burns, which may reflect the weight-bearing stance of koalas, given their syndactyly of digits II and III [14]. For K21-057, palmar burns were of a lesser grade, being similarly scored as superficial at necropsy and Grade I histologically. This could reflect the changes between the bipedal and quadrupedal stance of koalas for climbing and walking, respectively [15].

The distal digits and claws were examined radiologically and histologically based on abnormalities of the claws noted at necropsy, such as blunted claw tips that appeared to be ‘melted’ and erythema. Anecdotally, claws are often lost in rehabilitating koalas in the weeks and months following footpad burns, and blunted or melted ends of claw tips have previously been observed (author obs.). Claw loss in burnt koalas mirrors that which occurs in other species, such as the loss of hooves in burnt livestock [8]. In K21-047, there were radiographically lytic and sclerotic changes to the distal phalanges in those digits noted to be abnormal at necropsy, supporting the presence of heat radiation damage to the underlying ungual process and more proximal bone. However, these digits were not examined histologically, and in K21-050, in which one claw was lost from the ungual process of left fore digit IV, there was only soot and superficial secondary infection present. Given that these koalas were euthanised within the week following the fire, it may be that clinically observable changes due to damage to the nailbed of the claws occur at a later stage. In humans, osteolytic changes occur over many days to weeks. As such, the full extent of radiographic changes may not have been observed as resorptive processes predominate over approximately the first two weeks following an acute burn injury [16]. For K21-050, a distal phalangeal fracture in left hind digit V could also indicate that a fall or other traumatic event could have contributed to the claw loss.

Consistent findings from lung tissue were pulmonary oedema and congestion, possibly due to distal migration of upper airway material and the actions of systemic inflammatory mediators (mainly neutrophils) causing damage to the lower respiratory tract when hot air and smoke is inhaled [4,5,17]. Koalas have been reported to show variable clinical signs with respiratory disease [13], which could be explained by their more sedentary lifestyle [12]. Despite this, the extent of the pulmonary oedema present in the koalas in the current study show that this is a significant comorbidity that needs to be recognised by treating veterinarians.

Previous studies in other animal species have shown that burn wounds to the skin covering less than 20% of the total body surface area (TBSA) can be regarded as local burns, and patients with more than 20% are more likely to develop life-threatening metabolic disturbances. Burns that encompass more than 50% are considered incompatible with survival [6]. This method, along with assessment of the depth of burn, enables the veterinarian to predict prognosis and decide on the next steps for either treatment and rehabilitation or euthanasia [4]. However, in koalas, considering that palmar and plantar burns could cause potential catastrophic loss of function with regard to climbing trees for food, the application of the current TBSA method to burn assessment may not be as useful in formulating prognosis (only <10% TBSA if all four feet are burnt). Likewise, burns to the face (eyes, nose, lips) would result in difficulty feeding and intense discomfort. Hence, a koala-specific TBSA chart that gives greater weight to regions of the face and palmar and plantar surfaces is needed. In the future, the assessment of koala fire victims could then be improved in regard to the depth of the burn and the location of injury in relation to TBSA.

## 5. Conclusions

This study identified that cutaneous burn lesions in koalas may be more severe than is apparent upon clinical examination, in both the furred regions of the body as well as the palmar and plantar surfaces (footpads). The plantar surfaces were most commonly affected, particularly on the lateral aspect, and this may extend to the distal phalanges, resulting in claw loss due to heat damage. A koala-specific chart for TBSA calculation for koalas that emphasises the importance of palmar/plantar surfaces is much needed, and if used in conjunction with assessment of burn depth, should improve veterinarians’ ability to predict prognosis for koalas. The likelihood of concurrent pulmonary oedema should also be considered alongside visible burn lesions in the assessment of koalas in the triage and rehabilitation phases.

## Figures and Tables

**Table 1:** Details of four koalas rescued from bushfires.

Koala	Sex	Tooth Wear Class [12]	Approximate Age [12]	Body Condition Score [13]	Weight (kg)
K21-047	M	IV	5–6 years	3	9.7
K21-048	F	VI	12+ years	2.5	8.2
K21-050	F	II	2–3 years	3.5	7.5
K21-057	F	VI	12+ years	3.5	7.8

**Table 2:** Clinical and histopathologic assessments of sampled skin burn lesions.

Koala	Lesion Site	Clinical Assessment	Histopathologic Grade
K21-047	Pinna margins	Superficial thickness	III
	Dorsal skin over iliac crest	Superficial to partial thickness	IIa–III
	Right lateral plantar surface	Superficial to partial thickness	III
K21-048	Left lateral plantar surface	Superficial to partial thickness	III
K21-050	Left central plantar surface	Superficial to partial thickness	IIb–III
K21-057	Right palmar surface	Superficial thickness	I

## Data Availability

All data presented.
